# Progression and Outcomes of Non-dysfunctional Bicuspid Aortic Valve: Longitudinal Data From a Large Korean Bicuspid Aortic Valve Registry

**DOI:** 10.3389/fcvm.2020.603323

**Published:** 2021-01-11

**Authors:** Shinjeong Song, Jiwon Seo, Iksung Cho, Geu-Ru Hong, Jong-Won Ha, Chi Young Shim

**Affiliations:** ^1^Division of Cardiology, Department of Internal Medicine, Ewha Womans University Hospital, Ewha Womans University College of Medicine, Seoul, South Korea; ^2^Division of Cardiology, Severance Cardiovascular Hospital, Yonsei University College of Medicine, Seoul, South Korea

**Keywords:** bicuspid aortc valve, valve function, aortopathy, progression, outcomes

## Abstract

**Background:** Using echocardiographic surveillance, many patients are diagnosed with bicuspid aortic valve (BAV) without significant valve dysfunction. Limited data are available regarding the progression and outcomes of non-dysfunctional BAV.

**Methods and Results:** We investigated 1,307 BAV patients (984 male, mean age 56 years) diagnosed from Jan 2003 through Dec 2018 in a single tertiary center. Seven hundred sixty-one patients underwent follow-up echocardiography at ≥1 year post-diagnosis. Non-dysfunctional BAV was defined as BAV without moderate aortic stenosis (AS) or aortic regurgitation (AR). The presence of aortopathy was defined as an ascending aorta diameter >37mm. Progression to significant BAV dysfunction, progression to severe aortopathy (ascending aorta diameter ≥45mm), and incidence of valve or aorta operation were analyzed. One hundred eighty-seven (25%) patients showed non-dysfunctional BAV. Among them, 104 (56%) had mild AS or AR, and 81 (43%) had aortopathy at indexed echocardiography. At 6.0 ± 3.8 years post-diagnosis, 56 (29%) progressed to dysfunctional BAV, 28 (15%) progressed to severe aortopathy, 22 (12%) underwent valve operation, and 19 (10%) experienced aorta operation. Eighty-nine percent of patients with normal BAV function and 61% of patients with mild AS or AR maintained non-dysfunctional BAV. More patients with aortopathy progressed to severe aortopathy (35 vs. 0% without aortopathy, *p* < 0.001), with a higher incidence of aorta operation (21 vs. 2%, *p* < 0.001).

**Conclusions:** In patients with non-dysfunctional BAV, initial BAV function and degree of aorta dilatation might be important for progression and outcomes. Patients without any dysfunction or aortopathy tend to maintain good structure and function for 6 years.

## Introduction

Bicuspid aortic valve (BAV) is known as the most common congenital heart valve disease. Patients with BAV exhibit significant heterogeneity in various clinical aspects, including the type and degree of valve dysfunction or aortopathy (1–3). As echocardiographic surveillance has recently been carried out in the general population, the diagnosis of non-dysfunctional BAV, in which BAV has no significant aortic stenosis (AS) or aortic regurgitation (AR), is increasing.

It is well-established that patients with clinically significant AS or AR incur serious outcome consequences, whether they have bicuspid or tricuspid valves (4, 5). However, limited data are available regarding patients with normally functioning or minimally dysfunctional BAV at initial diagnosis (6, 7).

Our objective was to determine the incidence of aortopathy at initial diagnosis and characterize aortic complications among patients with non-dysfunctional BAV compared with dysfunctional BAV. We also used a large Korean BAV registry to assess the progression of valvular dysfunction and aortopathy in patients with non-dysfunctional BAV.

## Methods

### Study Population

We retrospectively reviewed the echocardiographic database and medical records of patients with BAVs diagnosed from January 2003 to December 2018 at Severance Cardiovascular Hospital (Yonsei University College of Medicine, Seoul, South Korea). During this period, 1,307 patients with BAVs were identified and included in our BAV registry. Among them, 761 patients had undergone follow-up echocardiography at a minimum of 1 year post-diagnosis.

**Figure 1:**
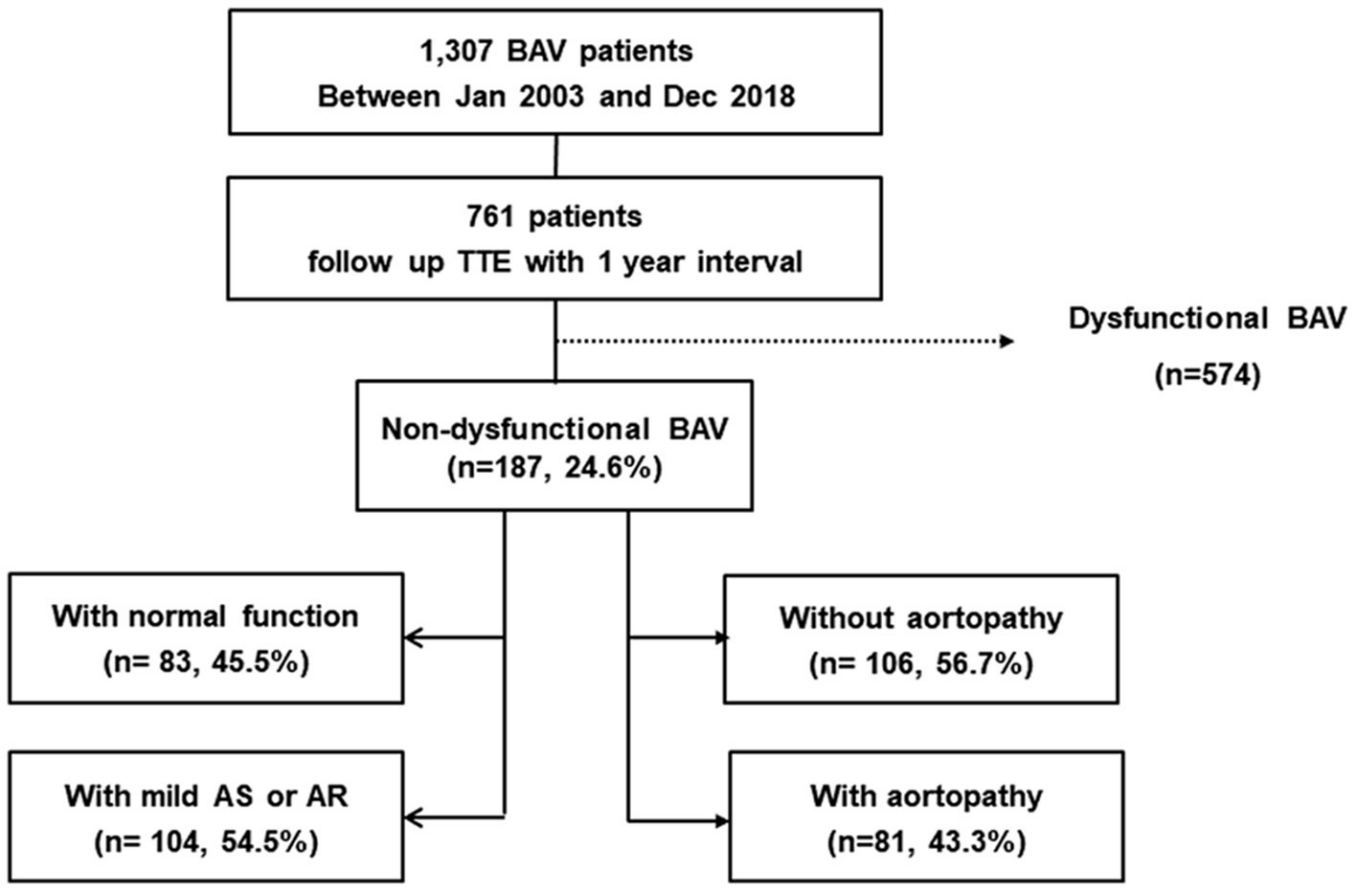
Flow chart of the study.

Significant AS and significant AR were detected via transthoracic echocardiograms; significant AS was defined as at least moderate AS, and significant AR was defined as at least moderate AR, using the guidelines in place (8, 9). Non-dysfunctional BAV was defined as BAV without significant AS or AR. Presence of aortopathy was defined as an ascending aorta (AA) diameter >37 mm (10, 11). We excluded 574 patients with significant AS or AR at the indexed echocardiogram in this study. Among 574 patients with dysfunctional BAVs, 354 showed severe AS or severe AR. During the mean follow-up of 5.9 years, 409 (71%) patients underwent operations (281 isolated BAV operation, 122 both BAV and aorta surgery and six isolated aorta surgery). One hundred eighty-seven non-dysfunctional BAV patients were classified according to valve function and aortopathy. For valve function, the patients were divided into the normal valve function group and the mild AS or AR group. In addition, they were divided into two groups according to presence of aortopathy (Figure 1).

The Institutional Review Board of Severance Hospital approved the present study, which was conducted in compliance with the Declaration of Helsinki.

### Echocardiographic Assessments

Standard two-dimensional and Doppler measurements were performed following the current guidelines (12). A congenital BAV was diagnosed when only two cusps were unequivocally identified in systole and diastole in the short-axis view, with a clear “fish mouth” appearance during systole, as previously described (13). Anatomical types of BAV were identified according to a classification system suggested by Schaefer and colleagues (14). Type 1 exhibits congenital fusion of the right and left coronary cusp. Type 2 has a congenital fusion of the right and non-coronary cusp. Type 3 exhibits a congenital fusion of the non-coronary and left coronary cusp. Type 0 has no raphe and is also called “true type BAV.” The severity of AS or AR was assessed using an integrated approach (9, 15). All measurements of the aorta were performed according to recommendations on the QRS complex of the electrocardiogram (12). The dimensions of the Valsalva sinuses were measured perpendicularly to the right and left (or non-) aortic sinuses. The sinotubular junction was measured where the aortic sinuses met the tubular aorta. The AA was measured ~2 cm distal to the sinotubular junction, as described previously (13). Echocardiographic data were gathered and analyzed by experienced echocardiographers who were blinded to each patient's clinical data.

### Statistical Analysis

Continuous variables are expressed as a mean ± standard deviation. Categorical variables are expressed as a number (percentage). Comparisons between groups were performed using standard chi-square tests for categorical variables and student *t*-tests for continuous variables. Multiple regression analysis was performed to determine the association between clinical and echocardiographic variables at the initial diagnosis and progression to BAV dysfunction or receiving aortic valve surgery. Similarly, multiple regression analysis was applied to find factors associated with the progression to severe aortopathy or receiving aorta surgery. The variables selected for entry into the multivariate analysis were those with a *p*-value <0.1 in the univariate analysis as well as other important variables. All statistical analyses were performed using SPSS Statistics, version 23.0 (IBM, Armonk, NY, USA). *P*-values <0.05 were considered statistically significant.

## Results

### Baseline Characteristics According to Valve Function and Aortopathy

Among 761 BAV patients in the registry, 187 (25%) patients showed non-dysfunctional BAV. Among these, 104 (56%) patients had mild AS or AR, and 81 (43%) had aortopathy at indexed echocardiography. The baseline characteristics of the subjects according to their baseline aortic valve function or presence of aortopathy were largely comparable (Table 1). Patients with aortopathy had a higher mean age and a higher use rate of beta blockers than those without aortopathy. However, the distribution of comorbidities, including hypertension, was similar between comparison groups. The most common BAV morphology was the type 1 morphology of fusion of the left and right coronary cusps in all groups, and the patients with aortopathy revealed a higher incidence of type 0 morphology than those without aortopathy (27.2 vs. 14.2%, *p* = 0.019) (Table 2). Patients with aortopathy showed a significantly larger aorta dimension than the other groups. Also, patients with aortopathy revealed a lower e' velocity than those without aortopathy.

**Table 1:** Baseline characteristics.

	**Normal** **function** **(*n* = 83)**	**Mild AS** **or AR** **(*n* = 104)**	**Without** **aortopathy** **(*n* = 106)**	**With** **aortopathy** **(*n* = 81)**
Age, y	52 ± 13	55 ± 13	51 ± 13	58 ± 11^†^
Male sex	64 (74.4)	81 (77.9)	82 (74.5)	63 (77.8)
BMI, kg/m^2^	24.4 ± 3.3	23.8 ± 3.1	23.8 ± 3.0	24.5 ± 3.5
Systolic BP, mmHg	125.9 ± 17.8	126.0 ± 16.2	127.0 ± 17.6	124.4 ± 16.1
Diastolic BP, mmHg	78.6 ± 11.4	79.0 ± 13.0	78.6 ± 12.3	79.1 ± 12.2
Comorbidities				
Hypertension	38 (44.7)	52 (50)	54 (49.1)	36 (44.4)
Diabetes mellitus	23 (27.1)	22 (21.2)	27 (24.5)	18 (22.2)
Chronic kidney disease	4 (4.6)	8 (7.7)	9 (8.2)	3 (3.7)
Dyslipidemia	34 (40.0)	30 (28.8)	36 (32.7)	28 (34.6)
Coronary artery disease	26 (30.6)	23 (22.1)	28 (25.5)	21 (25.9)
Medications				
RAAS blocker	27 (33.3)	32 (30.8)	29 (26.4)	30 (37.0)
Beta blocker	20 (24.7)	21 (20.2)	17 (15.5)	24 (29.6)^†^
Calcium channel blocker	19 (23.5)	23 (22.1)	27 (24.5)	15 (18.5)
Diuretics	10 (13.3)	15 (14.4)	31 (28.2)	13 (16.0)
Statin	25 (30.9)	26 (25.0)	12 (10.9)	20 (24.7)

*Data are shown as Mean ± SD or n (%)*.

P < 0.05 ^*^ compared with the normal function group,

† *compared with the group without aortopathy*.

*AS, aortic stenosis; AR, aortic regurgitation; BP, blood pressure; RAAS, renin angiotensin aldosterone system*.

**Table 2:** Echocardiogram characteristics.

	**Normal** **function** **(*n* = 83)**	**Mild AS** **or AR** **(*n* = 104)**	**Without** **aortopathy** **(*n* = 106)**	**With** **aortopathy ** **(*n* = 81)**
BAV morphology				
Type 1	48 (57.8)	69 (66.3)	69 (65.1)	48 (59.3)
Type 2	14 (16.9)	17 (16.3)	20 (18.9)	11 (13.6)
Type 3	1 (1.2)	1 (1.0)	2 (1.8)	0 (0)
Type 0	20 (24.1)	17 (16.3)	15 (14.2)	22 (27.2)^†^
BAV function				
Normal	83 (100)	0 (0) ^*^	50 (47.2)	33 (40.7)
Mild AR	0 (0)	51 (49.0)^*^	31 (29.2)	20 (24.7)
Mild AS	0 (0)	36 (34.6)^*^	17 (16.0)	19 (23.5)
Mild ASR	0 (0)	17 (16.3)^*^	8 (7.5)	9 (11.1)
Aorta dimension, mm	39.3 ± 5.8	38.5 ± 5.1	32.4 ± 3.5	44.3 ± 6.3^†^
Annulus, mm	20.0 ± 3.3	20.8 ± 3.3	19.3 ± 2.6	21.9 ± 3.7^†^
Sinus of Valsalva, mm	34.3 ± 6.4	34.0 ± 9.7	32.0 ± 5.7	36.8 ± 6.6^†^
Sinotubular junction, mm	30.1 ± 6.0	30.2 ± 5.1	27.6 ± 3.4	33.6 ± 6.0^†^
LVEDD, mm	49.7 ± 5.9	50.7 ± 6.1	49.6 ± 5.9	51.2 ± 6.1
LVESD, mm	33.7 ± 5.7	33.8 ± 7.0	33.1 ± 6.4	34.6 ± 6.3
LVEF, %	63.1 ± 9.1	64.5 ± 10.2	65.0 ± 8.9	62.7 ± 10.7
LV mass index, g/m^2^	92.8 ± 27.0	99.6 ± 28.8	95.9 ± 24.8	99.4 ± 32.4
LA volume index, ml/m^2^	25.0 ± 10.7	29.1 ± 14.0	28.6 ± 13.9	25.5 ± 10.7
e' velocity, cm/s	7.1 ± 2.4	6.8 ± 2.4	7.5 ± 2.4	6.2 ± 2.2^†^
S' velocity, cm/s	7.1 ± 1.5	7.0 ± 1.5	7.1 ± 1.6	6.9 ± 1.4
E/e'	10.6 ± 4.9	10.7 ± 4.7	10.5 ± 4.5	11.1 ± 5.2
RVSP, mmHg	24.8 ± 6.2	24.5 ± 5.5	24.7 ± 6.3	24.6 ± 5.1

*Data are shown as Mean ± SD or n (%)*.

P < 0.05

* *compared with the normal function group*,

† *compared with the group without aortopathy*.

*AS, aortic stenosis; AR, aortic regurgitation; BAV, bicuspid aortic valve; ASR, aortic stenosis with regurgitation; LVEDD, left ventricular end-diastolic dimension; LVESD, left ventricular end-systolic dimension; LVEF, left ventricular ejection fraction; LV, left ventricle; LA, left atrium; e', early diastolic mitral annular; S', systolic mitral annular; E/e', the ratio of early diastolic mitral inflow and early diastolic mitral annular; RVSP, right ventricular systolic pressure*.

### Progression to BAV Dysfunction and Incidence of Aortic Valve Operation

**Figure 2:**
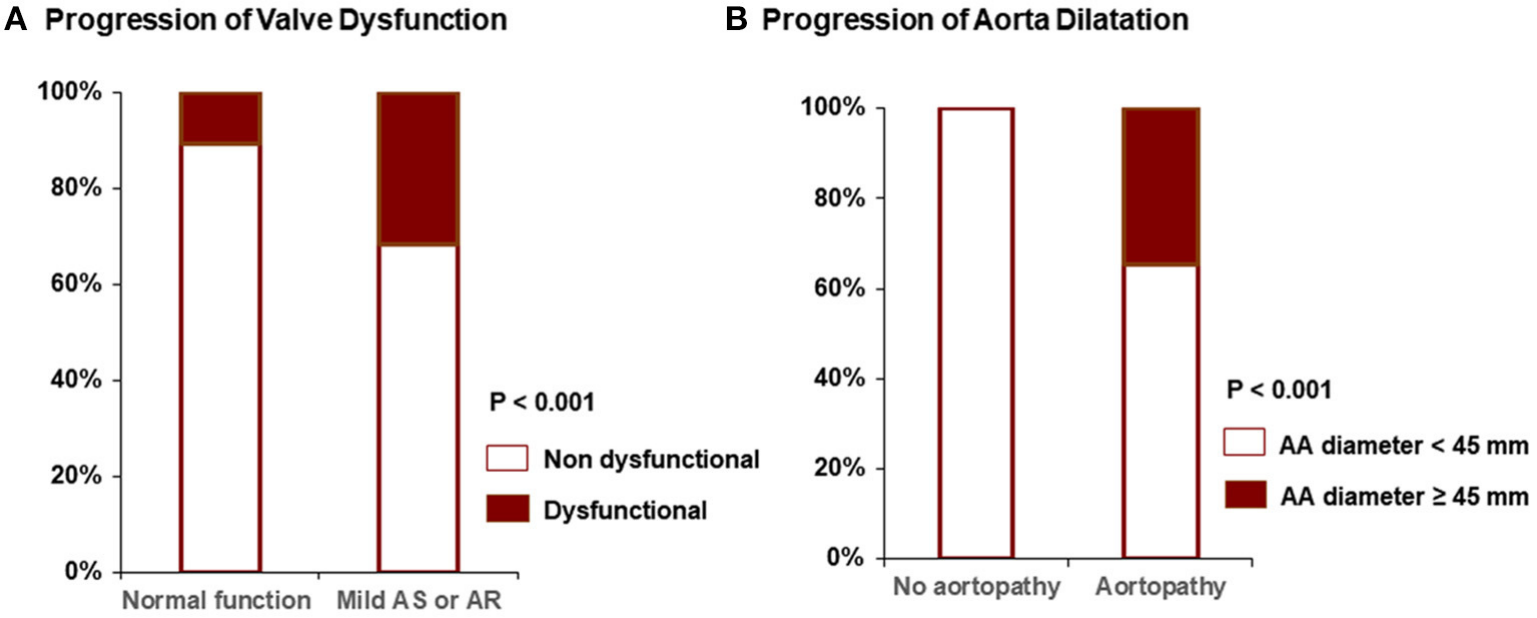
Progression to valve dysfunction **(A)** and aorta dilatation **(B)** between groups.

**Figure 3:**
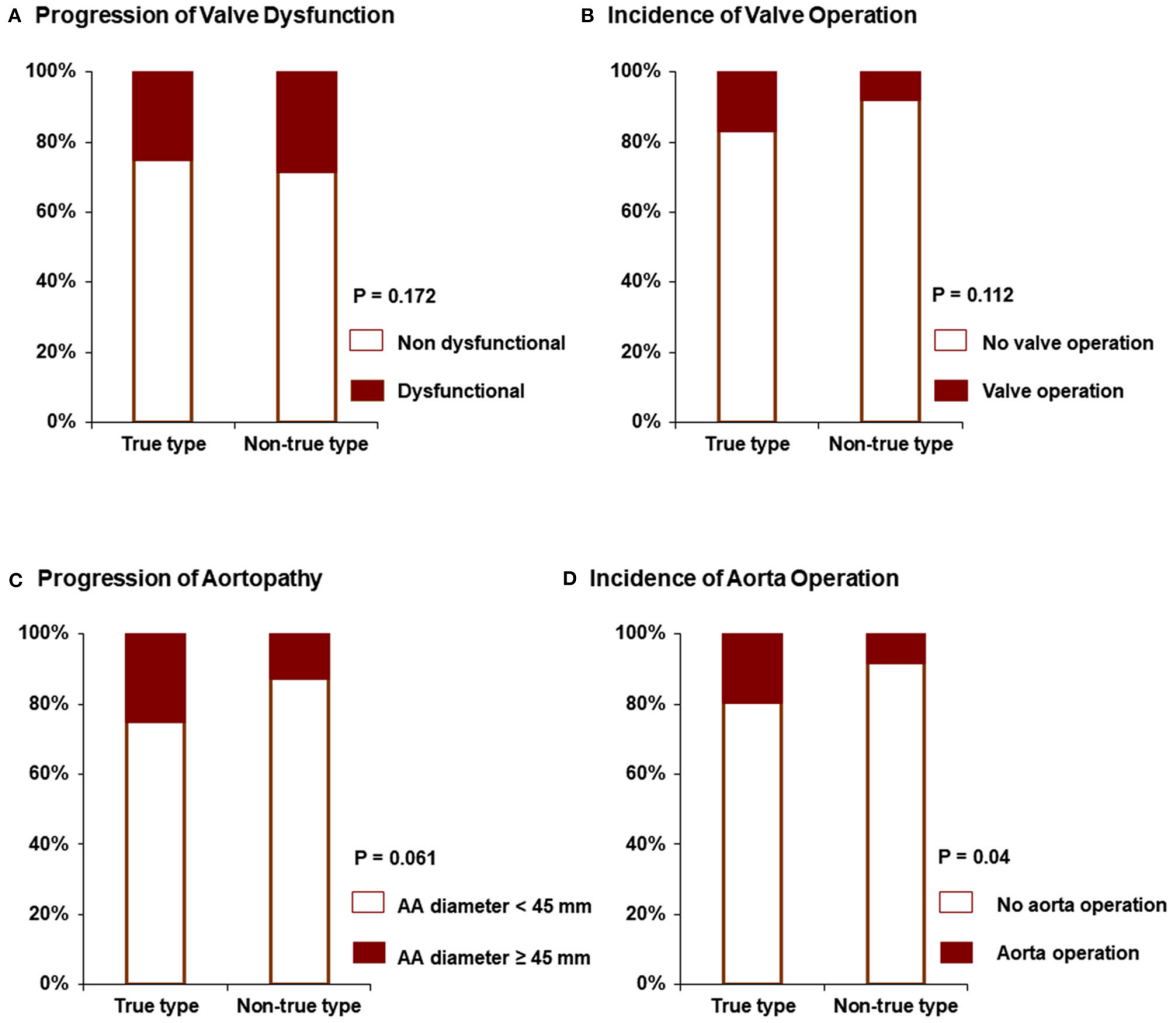
Progression to valve dysfunction **(A)**, the incidence of valve operation **(B)**, the progression to severe aortopathy **(C)**, and incidence of aorta operation **(D)** according to BAV morphology.

In the normally functioning BAV group, 87% maintained non-dysfunctional BAV after follow-up (mean follow-up duration: 5.8 yrs). However, in the group with mild AS or AR, 61% did not show progression to significant valve dysfunction (mean follow-up duration; 6.2 yrs) (Figure 2A). The follow-up echocardiographic characteristics and detailed information for the operation were presented in Supplementary Tables 1 and 2. Also, aortic valve operation tended to be more frequent in the group with mild AS or AR, but this did not meet statistical significance (12.5 vs. 6.0%, *p* = 0.068) (Table 3). In the analysis for progression of valve dysfunction according to BAV morphology, the ratio of progression maintained with non-dysfunction was similar regardless of the presence of true type BAV (75 vs. 72%, *p* = 0.172) (Figure 3A). However, true type BAV showed a tendency of a higher incidence of valve operation than other types (19 vs. 10%, *p* = 0.112) (Figure 3B). In multivariate analysis, the presence of mild AS and initial aorta dimension were independently associated with the progression to non-dysfunctional BAV or receiving aortic valve operation (Table 4).

**Table 3:** Progression of BAV dysfunction and incidence of aortic valve operation.

	**Normal function** **(*n* = 83)**	**Mild AS or AR** **(*n* = 104)**
Progression to dysfunctional BAV		
Moderate dysfunction	5 (6.0)	24 (23.1)^*^
Severe dysfunction	1 (1.2)	4 (3.8)
Aortic valve operation, *n* (%)	5 (6.0)	13 (12.5)
Severe dysfunction at operation	3 (3.6)	5 (4.8)
Non-severe dysfunction at operation	2 (2.4)	8 (5.7)
Coronary artery bypass graft	0 (0)	1 (0.9)
Graft replacement of ascending aorta	2 (2.4)	7 (6.7)
Follow-up duration, years	5.8 ± 3.6	6.2 ± 3.9

*Data are shown as Mean ± SD or n (%)*.

* *P < 0.05 compared with the normal function group*.

*AS, aortic stenosis; AR, aortic regurgitation; BAV, bicuspid aortic valve*.

**Table 4:** Factors associated with the progression to non-dysfunctional BAV or receiving aortic valve operation.

***R* = 0.438**	**β**	**T**	***P*-value**
Age	−0.075	−0.926	0.356
Male sex	−0.077	−1.068	0.287
Hypertension	0.041	0.498	0.619
Diabetes mellitus	0.039	0.493	0.623
Chronic kidney disease	−0.004	−0.047	0.962
RAAS blocker use	−0.027	−0.328	0.744
Beta blocker use	−0.041	−0.515	0.607
Statin use	0.014	0.177	0.860
Presence of mild AR	0.046	0.641	0.523
Presence of mild AS	0.305	4.228	<0.001
True type BAV	−0.054	−0.748	0.456
Initial aorta dimension, mm	0.314	3.997	<0.001

*RAAS, renin angiotensin aldosterone system; AR, aortic regurgitation; AS, aortic stenosis, BAV, bicuspid aortic valve*.

### Progression to Severe Aortopathy and Incidence of Aorta Operation

Patients with BAV were subdivided into two groups according to the presence of aortopathy. Compared to patients without aortopathy, the aortopathy group showed a tendency for faster progression, but this was not statistically significant (0.42 ± 0.85 mm vs. 0.32 ± 0.66 mm, *p* = 0.22) (Table 5). None of the patients without aortopathy experienced a progression of >45 mm in aorta diameter during follow-up (mean 6.0 years), whereas 34.6% of patients with aortopathy had an increase in aorta diameter of >45 mm (Figure 2B). Furthermore, 9.9% of the patients with aortopathy had an increase of >50 mm. Also, in the group with aortopathy, the rate of surgery was significantly higher (21.0 vs. 1.8% without aortopathy, *p* < 0.001) during the follow-up period (mean 6.0 years). The progression of aortopathy was analyzed according to BAV morphology. The rate of progression to severe aortopathy or the rate of aorta operation was higher in true type BAV than in other types of BAV (Figures 3C,D). However, in multivariate analysis, initial aorta dimension was the single independent predictor for the progression to severe aortopathy or receiving aorta operation (Table 6).

**Table 5:** Progression of aortopathy and incidence of aorta operation.

	**Without aortopathy** **(*n* = 106)**	**With aortopathy** **(*n* = 81)**
Progression rate, mm/year	0.32 ± 0.66	0.42 ± 0.85
Initial AA diameter, mm	32.3 ± 3.5	42.0 ± 6.3^*^
Final AA diameter, mm	34.1 ± 3.6	44.0 ± 5.1^*^
Progression to severe aortopathy		
AA diameter ≥ 45 mm	0	28 (34.6)^*^
AA diameter ≥ 50 mm	0	8 (9.9)^*^
Aorta operation	2 (1.8)	17 (21.0)^*^
Follow-up duration, years	6.0 ± 3.5	6.1 ± 4.1

*Data are shown as Mean ± SD or n (%)*.

* *P < 0.05 compared with the group without aortopathy*.

*AA, ascending aorta*.

**Table 6:** Factors associated with the progression to severe aortopathy or receiving aorta operation.

***R* = 0.782**	**β**	**T**	***P*-value**
Age	−0.006	−0.114	0.909
Male sex	−0.30	−0.606	0.545
Hypertension	−0.076	−1.320	0.189
Diabetes mellitus	0.012	0.226	0.821
Chronic kidney disease	−0.007	−0.144	0.886
RAAS blocker use	−0.065	−1.134	0.258
Beta blocker use	0.078	1.397	0.164
Statin use	−0.054	−1.000	0.319
Presence of mild AR	−0.064	−1.270	0.206
Presence of mild AS	0.022	0.445	0.657
True type BAV	−0.007	−0.140	0.889
Initial aorta dimension, mm	0.749	13.724	<0.001

*RAAS, renin angiotensin aldosterone system; AR, aortic regurgitation; AS, aortic stenosis; BAV, bicuspid aortic valve*.

## Discussion

The principal findings in the present study are that (1) aortopathy was quite common in patients with BAV, even in the absence of significant valvular dysfunction, (2) the progression of AA dilatation that met the requirements for aorta operation was more common in BAV patients with aortopathy at initial diagnosis than in those without aortopathy, (3) 89% maintained non-dysfunctional BAV after 6 years of follow-up when BAV patients were diagnosed with normal valve function. However, only 61% maintained non-dysfunctional BAV in patients with mild dysfunction, and (4) the rate of progression to severe aortopathy or the rate of aorta operation was higher in true type BAV than in other types of BAV.

The ratio of aortopathy to valvulopathy varies in patients with BAVs. The results of the present study also allow for the interpretation that the proportion of aortopathy is significant in the absence of valvulopathy (16–19). Our findings support previous studies suggesting that BAV is associated with intrinsic aortopathy, as well as with valve function-related pathology (16–19). We found that the association with the initial degree of BAV aortopathy was important in determining the incidence of aorta operation. Interestingly, when patients with initial normal valve function with advanced aortopathy (>45 mm, 28 patients) were followed up for 6.1 years, 14 (50.0%) patients underwent aorta and valve surgery. Four of the 14 patients underwent aortic valve replacement and aorta surgery for severe AS or severe AR, and 10 patients underwent aortic valve replacement with aorta operation, even though BAV function was normal or mildly dysfunctional. Thus, the aortopathy predominant patients with non-dysfunctional BAVs experienced aortopathy-associated clnical events during about 6 years. Because there was no adequate information on the natural history of existing BAV, additional aortic valve replacement was considered. However, according to our study, overall 71% of patients with non-dysfunctional BAVs maintained non-dysfunctional BAVs at 6 years follow-up. In young female patients of childbearing age, warfarin would be indicated for a long time in operations performed with mechanical valves. Therefore, when performing aorta operation in patients with non-dysfunctional BAVs, the decision of concomitant aortic valve replacement should be made cautiously in considering the individual's risk and benefits.

In general, degenerative changes in BAV patients occur earlier than in tricuspid AV patients. Recently, the diagnosis of normally functioning BAV in patients without valve dysfunction and aortopathy is increasing. There have been studies on factors that determine valve dysfunction in BAV patients (20–22) or progression in BAV patients with significant valvular dysfunction (4). Previous studies have also examined how these factors affect left ventricular diastolic function, according to BAV morphology (13). Moreover, a previous report from the Korean BAV cohort also demonstrated mid-term clinical outcome in asymptomatic or mildly symptomatic patients with BAVs including both non-dysfunctional BAVs and dysfunctional BAVs (23).

However, there have been few studies on the natural course of normally functioning BAV patients, and in the real world, patients may wonder about their prognosis and when to perform a follow-up echocardiogram. As a result, we expect our research to serve as a reference. Although the mean follow-up duration was not long enough (about 6 years), ~89% of patients with normal valvular functional BAV at the time of diagnosis had no surgical treatment during the follow-up period, and 71% of the patients maintained mild valve dysfunction during the follow-up period. Even for non-dysfunctional BAV patients, if the aorta is over 37 mm at the time of diagnosis, or if there is mild BAV dysfunction, if true type BAV, the progression of aortopathy or BAV dysfunction should be regularly examined by echocardiography.

Studies of BAV have increased rapidly during recent years. An international BAV consortium has identified knowledge gaps and risen to the challenge regarding BAV (24). Also, the American Association for Thoracic Surgery published consensus guidelines on BAVs (25). Genetic studies on BAV have been published, some groups have broadened the scope of transcatheter aortic valve replacement to focus on BAV (26). However, few natural history data are based on long-term observations. In particular, the Olmsted county study is an ideal community-based study, whereas our BAV registry is affected by several sources of bias because we included a referral cohort. The patients in the Olmsted County study were obtained by screening through auscultation revealed that 27% had aortic valve- or aorta-related surgery within a 20-year follow-up period (27). Similar to our registry, Olmsted's study also constructed a cohort in normal or mild aortic valve disease patients. In Olmsted study, the results for detailed follow up-echocardiography were missing, the rate of surgery was shown. Compared to the olmsted study, it is noteworthy that the rate of surgery of our study after mean 6 years follow-up is similar. In comparison, our study has the advantage of including detailed echocardiographic follow-up data for valve function and aorta dimensions as well as clinical outcomes over 6 years in patients with non-dysfunctional BAV. The present study provides additional information to help clinicians predict which patients will progress and worsen clinical outcomes.

## Limitations

The present study had several limitations. First, this retrospective study included only Korean BAV subjects from a single tertiary referral center, which may result in bias. Therefore, multi-center, prospective studies are needed to evaluate the prevalence of aortopathy in normal valvular BAV and progression of aortic valve function in BAV. Since the follow-up period for valve dysfunction is different for each patient, it is limited in its use for quantitative evaluation of the progression of valve dysfunction. However, we believe that this study is a meaningful study that has reported on the prevalence of aortopathy and valve progression in a large Korean registry using comprehensive reviews. Additionally, the median follow-up duration was only 6 years, which is insufficient to analyze the long-term natural history of early BAV disease. Second, data were lacking regarding common genetic backgrounds in BAV patients. Third, aortic diameters were measured based on echocardiographic imaging alone, because only some BAV subjects underwent computed tomography or cardiac magnetic resonance imaging.

## Conclusions

In patients with non-dysfunctional BAV, initial BAV function and degree of aorta dilatation might be important for progression and outcomes. Patients without any dysfunction or aortopathy tend to maintain good structure and function for 6 years.

## Data Availability Statement

The original contributions presented in the study are included in the article/Supplementary Material, further inquiries can be directed to the corresponding author/s.

## Ethics Statement

The studies involving human participants were reviewed and approved by Severance Hospital, Yonsei University College of Medicine. The ethics committee waived the requirement of written informed consent for participation.

## Author Contributions

SS and CS are the gurantors of the entire manuscript. CS and G-RH contributed to the study conception and design, critical revision of the manuscript for important intellectual content, and final approval of the version to be published. JS, IC, and J-WH contributed to the data acquisition, analysis, and interpretation. All authors contributed to the article and approved the submitted version.

## Conflict of Interest

The authors declare that the research was conducted in the absence of any commercial or financial relationships that could be construed as a potential conflict of interest.
