# Supplementary material for: Progression and Outcomes of Non-dysfunctional Bicuspid Aortic Valve: Longitudinal Data From a Large Korean Bicuspid Aortic Valve Registry
Source: Front Cardiovasc Med. 2021 Jan 11;7:603323. doi: 10.3389/fcvm.2020.603323 (PMC7829218; doi:10.3389/fcvm.2020.603323)
Supplement: Supplementary file 1 [file Table_1.DOCX]

| **Supplementary Table 1. Follow-up echocardiographic characteristics** | | | | |  |
| --- | --- | --- | --- | --- | --- |
|  | **Normal**  **function**  **(n= 83)** | **Mild**  **AS or AR**  **(n= 104)** | **Without aortopathy**  **(n= 106)** | **With**  **aortopathy**  **(n= 81)** | |
| BAV function |  |  |  |  | |
| AV PSPG, mmHg | 24.0±13.0 | 34.0±11.5* | 36.3±12.3 | 29.1±12.4 | |
| AV MSPG, mmHg | 12.5±7.4 | 18.8±7.1* | 18.5±5.8 | 16.4±8.3 | |
| Mild AR | 13 (15.7) | 51 (49.0)* | 37 (34.9) | 2 (33.3) | |
| Moderate AR | 0 (0) | 11 (10.5)* | 9 (8.5) | 2 (2.5) | |
| Severe AR | 1 (1.2) | 1 (0.9) | 0 (0) | 2 (2.5) | |
| Mild AS | 18 (21.7) | 39 (37.5)* | 30 (28.3) | 27 (33.3) | |
| Moderate AS | 5 (6.0) | 13 (12.5)* | 6 (5.7) | 12 (14.8) | |
| Severe AS | 0 (0) | 3 (2.9) | 3 (2.8) | 0 (0) | |
| Aorta dimension, mm | 39.3±8.8 | 39.1±7.0 | 34.2±3.9 | 45.7±6.7† | |
| LVEDD, mm | 49.1±6.2 | 49.5±5.6 | 49.2±6.0 | 49.4±5.7 | |
| LVESD, mm | 33.2±6.3 | 33.1±6.8 | 33.2±6.9 | 33.0±6.0 | |
| LVEF, % | 64.6±7.9 | 65.9±8.2 | 65.0±7.7 | 65.7±8.7 | |
| LV mass index, g/m^2^ | 92.3±26.9 | 96.4±22.7 | 94.3±25.5 | 97.1±23.7 | |
| LA volume index, ml/m^2^ | 28.9±10.5 | 30.3±10.6 | 30.0±11.9 | 29.2±8.7 | |
| e’ velocity, cm/s | 7.0±2.0 | 6.5±2.2 | 7.3±2.3 | 6.0±2.0† | |
| S’ velocity, cm/s | 6.9±2.0 | 6.8±1.4 | 7.1±1.9 | 6.5±1.4 | |
| E/e’ | 11.2±6.1 | 11.1±4.9 | 11.0±5.1 | 11.3±5.9 | |
| RVSP, mmHg | 25.4±6.1 | 26.6±7.3 | 26.1±6.9 | 26.1±6.8 | |

Data are shown as Mean ± SD or n (%).

P < 0.05 * compared with the normal function group, † compared with the group without aortopathy

AV indicates aortic valve; PSPG, peak systolic pressure gradient; MSPG, mean systolic pressure gradient; AS, aortic stenosis; AR, aortic regurgitation; LVEDD, left ventricular end-diastolic dimension; LVESD, left ventricular end-systolic dimension; LVEF, left ventricular ejection fraction; LV, left ventricle; LA, left atrium; e’, early diastolic mitral annular; S’, systolic mitral annular; E/e’. the ratio of early diastolic mitral inflow and early diastolic mitral annular; RVSP, right ventricular systolic pressure
