# Supplementary material for: Progression and Outcomes of Non-dysfunctional Bicuspid Aortic Valve: Longitudinal Data From a Large Korean Bicuspid Aortic Valve Registry
Source: Front Cardiovasc Med. 2021 Jan 11;7:603323. doi: 10.3389/fcvm.2020.603323 (PMC7829218; doi:10.3389/fcvm.2020.603323)
Supplement: Supplementary file 2 [file Table_2.DOCX]

**Supplementary Table 2. Types of operation during follow-up**

|  | **N = 187** |
| --- | --- |
| Patients who underwent operation | 23 (12.3) |
| Aortic valve and aorta surgery | 14 (7.5) |
| Aortic valve replacement and graft replacement of the aorta | 11 (5.9) |
| Aortic valve repair and graft replacement of the aorta | 2 (1.1) |
| Double valve replacement and graft replacement of the aorta | 1 (0.5) |
| Isolated aortic valve surgery | 3 (1.6) |
| Aortic valve replacement and CABG | 1 (0.5) |
| Isolated aorta surgery | 5 (2.7) |
| Graft replacement of the aorta | 3 (2.7) |
| Bypass surgery due to coactation | 2 (1.1) |

Data are shown as n (%).

CABG, coronary artery bypass graft
